# Molecular Physiological and Pathological Progression of Neurological Diseases

**DOI:** 10.3390/ijms26157084

**Published:** 2025-07-23

**Authors:** Daniele Bottai, Raffaella Adami

**Affiliations:** Department of Pharmacological Sciences, University of Milan, Via Giuseppe Balzaretti 9, 20133 Milan, Italy; raffaella.adami@unimi.it

Neurological diseases encompass a variety of conditions that vary in terms of when they first manifest, who is affected, what area of the nervous system is impacted, what type of neurons are damaged, whether they are genetically or spontaneously inherited, and whether they are curable.

Common neurological illnesses include autoimmune, traumatic, neurodegenerative, psychiatric, and cerebrovascular disorders. These diseases influence several molecular pathways and exhibit distinct pathophysiological characteristics and cellular changes that can disrupt many brain circuits. A variety of cellular and molecular characteristics that cannot be examined in patients can be evaluated using organoids and pluripotent stem cells; these characteristics are being studied in cellular models, including human-induced pluripotent stem cells and animal models.

There were fourteen Contributions published in this Special Issue.

Contributions 1 and 2 focused on multiple sclerosis (MS). MS is a severe neurological condition that results in inflammatory infiltrates, demyelination, astrogliosis, and early axonal damage due to an autoimmune response [1]. The experimental autoimmune encephalomyelitis (EAE) mouse model [2] is a popular and widely used animal model for studying MS; it resembles MS in a number of clinical, pathological, and immunological aspects, including inflammation, oligodendrocyte loss, demyelination, axonal loss, and neuronal loss.

Both MS and EAE have cytokine dysfunction [3]. In these two Contributions, the Sheikh F. Ahmad group determined that although using a histamine H4 receptor (HR4) antagonist was able to improve the disease’s outcome by modifying the balance of T-cells, the activation of the HR4 exacerbated the progression of MS by triggering a proinflammatory signal from B-cells. In conclusion, these two studies suggested that HR4 would be a good target for MS therapeutic interventions.

Spinal muscular atrophy (SMA) is another uncommon neurological condition that affects motor neurons. It is the most frequent of the rare genetic illnesses and primarily affects infants, young people, and newborns [4,5].

The authors of Contributions 3 and 4 conducted a joint investigation on muscle (Contribution 3) and neural stem cells (NSCs) (Contribution 4) derived from an animal model of SMA (SMN∆7 mice). The functional, biochemical, and molecular characteristics of isolated diaphragms in SMA are little understood. The data obtained from Contribution 3 point to the existence of a substantial build-up of reactive oxygen species (ROS) and an underlying energy imbalance linked to mitochondrial malfunction in the diaphragm. In turn, ROS buildup can lead to diaphragm atrophy, muscular fatigue, and eventually respiratory failure in SMN∆7 animals. The diaphragm recovers its functions after being exposed to the antioxidant ergothioneine, indicating the existence of redox imbalance and mitochondrial dysfunction. In Contribution 4, however, they showed that NSCs from the subventricular zone of SMA mice had reduced metabolic activity, clonogenic potential, and proliferation. This tendency was nearly reversed by the administration of curcumin, another antioxidant. These results raise the prospect of improving the disease’s prognosis in SMN∆7 mice by administering dietary supplements.

Epilepsy is a neurological condition marked by a propensity for epileptic seizures, neurobiological alterations, and cognitive, psychological, and social deficits [6]. The World Health Organization estimates that over 75 million individuals worldwide have been diagnosed with epilepsy. Through a comprehensive revisional study, the authors of Contribution 5 discovered that the alteration of circulating miR-181a is linked to alterations in protein, whereas circulating miR-21 and miR-155, are also altered during epilepsy, are linked to cell growth and death. These miRNAs participate in processes that may be indirectly linked to epileptogenesis, although they are not specialized for epilepsy. In another work, the impact of Levetiracetam (LEV), a novel anticonvulsive medication with an unclear mechanism, was examined (Contribution 6). This work aimed to ascertain how LEV affected the activity of four antioxidant enzymes in the hippocampus of an animal model of temporal lobe epilepsy (TLE): glutathione peroxidase, glutathione reductase, and superoxide dismutase (SOD). The findings showed that the epileptic group (EPI) had considerably higher SOD activity than the control group. The oxidant indicators of the groups did not differ significantly. The research in Contribution 6 indicates that LEV may alter the antioxidant/oxidant system in a time-dependent manner as epilepsy progresses, while further research is required to fully understand how LEV maintains redox equilibrium during chronic treatment.

Autism spectrum disorder (ASD) comprises a diverse collection of neurodevelopmental diseases, linked to limited and repetitive behavior as well as social–communication impairments. The frequency of ASD diagnoses has been rising significantly in recent decades. Male children are three times more likely than female children to have the disorder, with a current frequency of 1 in 36 children in the USA [7,8].

ASD’s precise etiology and pathology are yet unknown, although it is thought to be caused by a combination of environmental and genetic variables that contribute to immunological imbalance, oxidative stress, and mitochondrial malfunction [9]. Contribution 7’s finding offers further proof of particular immune cell bioenergetic profiles and increased inflammation-related chemicals in ASD. According to these outcomes, mitochondrial dysfunction is more prominent in ASD without inflammation and more significant in ASD with regression.

The Middle East and North Africa (MENA) area has a high level of genetic diversity and is underrepresented in genetic studies, particularly in autism research. The authors of Contributions 8 and 9 examined potential risk genes for autism in these populations. Historical migratory patterns, tribal heritage, and high consanguinity rates—which range from 20% to 50% throughout the Middle East, especially in the Arab Gulf countries—are some of the causes of this variety [10].

As detailed in Contribution 8, researchers identified 13 genes—including the ubiquitin pathway, solute transporters, chromatin remodelers, kinases, transcription factors, glutamate receptors, and ion channels—that were classified as possible risk genes in a Qatari cohort, emphasizing their functions in cognitive development. Contribution 9 presents the study of a cohort from Oman, identifying 48 genes that had not previously been linked to ASD and 35 genes that had previously been linked to neurodevelopmental disorders in populations worldwide. This work offers a thorough genetic analysis of ASD in the Omani community, providing a vital basis for comprehending the genetic intricacy of ASD in a consanguineous area.

It is speculated that hyperphosphorylated tau may lead up to and additionally boost filament formation, causing the production of these pathological inclusions. However, the exact mechanisms by which tau proteins (microtubule-associated proteins highly expressed in the central nervous system (CNS) [11] aggregate in the CNS are not fully understood [12].

The neuropathological features of Alzheimer’s disease (AD) are identified by Contribution 10’s description of two monoclonal tau antibodies that are produced against a peptide in the proline-rich region with three phosphorylation sites. These antibodies will be a useful tool for researching the biophysical characteristics of tau inclusions and their pathogenesis in related tauopathies. Contribution 11 presents an investigation into the effects of a soluble epoxide hydrolase (sEH) inhibitor on amyloid pathology, neurovascular coupling, blood–brain barrier integrity, neuroinflammation, neuron loss, and cognitive performance in a rat model of AD in an effort to find a novel therapy for the disease. In order to assess the expression profile of genes linked to inflammation, the authors of Contribution 12 examined cerebral small vessel disease, a prevalent condition with a significant socioeconomic cost that is characterized by a variety of forms and frequent comorbidity with AD. In addition to providing a comparatively thorough evaluation of the most recent therapeutic developments and emerging therapies for PD, Contribution 13 addresses the general elements of PD and compares the standard and innovative therapeutic approaches.

Spinal cord injury (SCI) is a debilitating medical illness that is commonly linked to significant morbidity and disability [13]. The authors of Contribution 14 aimed to determine whether alterations in T lymphocytes might worsen the course of chronic SCI and cause significant harm; in fact, the patients’ condition of T-lymphocytic immunodeficiency worsened during the course of the illness. The severity of these T cell deficits may be linked to the development of several immune-mediated illnesses, including metabolic and vascular disorders, the propensity to experience severe and recurring infections, and the poor response to immunizations.

A comprehensive knowledge of the molecular, cellular, and physiological components of neurological illnesses is required to create innovative therapeutic approaches that may prevent or treat these conditions. These therapies may concentrate on the many mechanisms that result in the healing of neurons and glia.

In conclusion, this Special Issue provides a broad comparative and integrative overview of disorders in terms of molecular processes and therapeutic approaches, due to the Contributions that can help, although partially, us to understand possible common pathways and similar actions of intervention.

## Data Availability

Data are available upon request.
